# Stanene cyanide: a novel candidate of Quantum Spin Hall insulator at high temperature

**DOI:** 10.1038/srep18604

**Published:** 2015-12-21

**Authors:** Wei-xiao Ji, Chang-wen Zhang, Meng Ding, Ping Li, Feng Li, Miao-juan Ren, Pei-ji Wang, Shu-jun Hu, Shi-shen Yan

**Affiliations:** 1 School of Physics and Technology, University of Jinan, Jinan, Shandong, 250022, P. R. China; 2 School of Physics, State Key laboratory of Crystal Materials, Shandong University, Jinan, Shandong, 250100, P.R. China

## Abstract

The search for quantum spin Hall (QSH) insulators with high stability, large and tunable gap and topological robustness, is critical for their realistic application at high temperature. Using first-principle calculations, we predict the cyanogen saturated stanene SnCN as novel topological insulators material, with a bulk gap as large as 203 meV, which can be engineered by applying biaxial strain and electric field. The band topology is identified by Z_2_ topological invariant together with helical edge states, and the mechanism is *s-p*_xy_ band inversion at G point induced by spin-orbit coupling (SOC). Remarkably, these systems have robust topology against chemical impurities, based on the calculations on halogen and cyano group co-decorated stanene SnX*x*X′1*−x* (X,X′  =  F, Cl, Br, I and CN), which makes it an appropriate and flexible candidate material for spintronic devices.

Topological insulator (TI) of two-dimensional (2D) materials, which has a semiconductor/insulator bulk state but a gapless edge state protected by time-reversal symmetry, is one kind of new quantum states of materials^1^,^2^,^3^. It is also called quantum spin Hall (QSH) insulator for the foundation of QSH effects caused by the spin-orbit coupling (SOC), characterized by insulating bulk gap and gapless edge states at boundaries due to time-reversal symmetry (TRS). Protected from backscattering in the edge state, it is possible for electrons to conduct without dissipation. In 2D materials, graphene is first identified to be TI^4^, but with a frustrating small SOC gap as small as 10^−3^ eV. After that, the researches on 2D TI with large gap become intensive.

Many efforts have been taken to find appropriate candidate compounds of large-gap 2D TI, whose bulk band gap could preserve at room temperature. Several single-layer honeycomb lattice, such as Bi^5^, Sb^6^, Ge^7^ and Sn^8^,^9^,^10^,^11^, have been studied deeply. For example, a low-buckled Sn honeycomb monolayer, which is called stanene, was predicted to be QSH insulators by Yao’s group^8^. Soon later, ultrathin stanene films with buckled configuration were observed experimentally by molecular beam epitaxy^10^,^11^.

In the study on 2D materials, the chemical modification (or functionalization) is considered to be an effective way to alter the electronic properties with desirable features and a powerful tool to find new materials with diverse possibilities, for the wide and various options in hundreds of chemical groups^12^,^13^,^14^,^15^,^16^,^17^. Taking 2D stanene as an example^9^, pure stanene has a bulk band gap of about 100 meV, while halogen decorated stanene, which remains topological nontrivial, could reach a gap of up to 300 meV. Cao *et al.*^18^ reported that halogeno decorated stanene presents phase transition between normal and topological phase with sizable gap under external strains. Motivated by these achievements, we are trying to find other topological candidates of stanene with different chemical groups decorated. Recently, organic groups decorated 2D structures have attracted more and more interests, as an alternative options to build novel TI materials. As is known, cyano group (-CN) has a similar chemical property to halogeno groups, hence cyanogen decorated stanene, if topological nontrivial, could be a diversified selection in spintronics devices.

Here we report on a novel 2D TI, cyano-group decorated stanene SnCN, based on first-principles calculations. The stability is confirmed by the calculations on formation energy and phonon spectrum. The topological characteristics are identified by calculating topological invariant 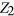, as well as their helical edge states. Furthermore, the topological properties of SnCN under bi-axial strains and their tunable band gap under vertical electric fields are investigated. Finally we try to explore the robust topological characters of co-decorated stanene against chemical impurities. We hope the organic group functionalizations on 2D films could provide new platforms for the designing of large gap QSH insulators.

## Results and Discussions

### Geometric and electronic structures of bulk material

**Figure 1:**
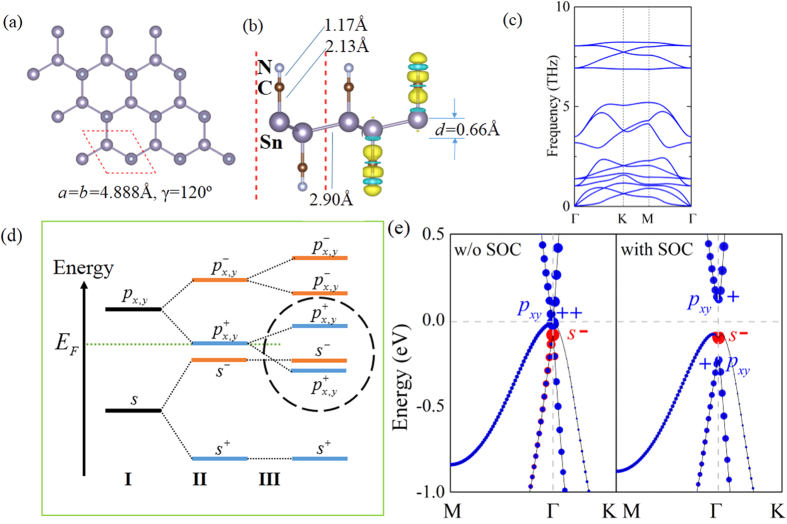
Geometric and electronic structures. (**a**) Top view of geometric structures of SnCN monolayer, with the lattice parameters of the unit cell. (**b**) Side view of geometric structures of SnCN monolayer, with the bond length, buckle distance and charge transfers between Sn atom and CN. Yellow and blue area denote the accumulation and depletion electrons respectively. (**c**) Phonon band dispersion relations. (**d**) Schematic diagram of the evolution from atomic *s* and 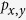 orbitals of Sn in SnCN into conduction and valence bands at Γ point. The three stages (I–III) represent the orbital orders with atomic potential, crystal field and spin-orbit coupling taken into account sequentially. The green dashed line denotes the Fermi energy 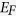. (**e**) Energy band structure near Fermi level without (left) and with (right) SOC interaction. Parities of Bloch states are denoted by +, −. The red/green dots denote *s*/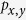 dominated orbitals. The size of dot denotes the weight of projection.

Similar to silicene^19^, stanene has a low-buckled configuration which is related to the relatively weak *π*-*π* bonding between Sn atoms. When decorated by cyano groups, SnCN prefers a stable *sp*^3^ configuration with the functional groups alternating on both sides of the stanene layer. Fully relaxed geometric structure of cyanogen decorated stanene SnCN is shown in Fig. 1(a,b). There is no mode with imaginary frequencies in its phonon spectrum, expecting its dynamical stabilities, see Fig. 1(c).

Compared to pure stanene^9^, the CN decorated statene has the Sn-Sn bond slightly increased, the buckling decreased and lattice constant enhanced, which is quite similar with halogenated stanene^9^. Figure 1(b) shows the charge redistribution after the decoration of cyano group, illustrating clearly the saturation of *p*_*z*_ orbits of Sn atoms. A detailed charge population is further calculated by Bader method^20^. The results show that each Sn loses one electron towards cyano group, from which the strong Coulomb interaction bonding -CN firmly on stanene film. However, this charge transfer has little effect on the Sn-Sn bond, as no charge redistribution between tin atoms before and after the adsorption of cyano group. It is also consist with the tiny change of the Sn-Sn bond length, by only 0.07 Å enhanced with cyano group. The high structural stability could be verified by the calculations of formation energy defined as


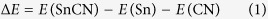


where 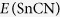 and 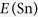 is the total energy of cyanogen functionalized and free-standing stanene respectively, while 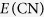 is the chemical potential of single −CN group. The formation energy shows the bonding between stanene film and CN, as large as −6.29 eV per CN, which is higher than the value of −5.86 eV in SnCl, indicating the stability of the bond between tin atoms and cyano groups.

Figure 1(e) illustrates the electronic band structures of the SnCN film at ground state, based on first-principles calculations without and with SOC. The even/odd parities of Bloch states are denoted by +/− at Γ point. The states around Fermi level are mainly contributed by 5*s* and 5*p*_*x, y*_ orbitals of Sn atoms, represented by red and blue dots in Fig. 1(e) respectively. And their 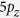 orbitals are saturated by cyano groups, as is shown in the charge redistribution in Fig. 1(b), leading to a large band gap at *K* point compared to free-standing stanene. Orbitals near Fermi level are labeled as 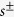 and 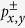, where the superscripts + and − denotes the parity. Without SOC taken into account, the system has the 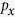 and 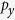 orbitals degenerate due to the 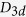 rotation symmetry of the configuration. The Fermi level is located in the bonding state of 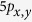 orbitals at Γ point, resulting as a zero-gap semiconductor, while the antibonding state of 5*s* is a little lower in energy, as is shown stage II of Fig. 1(d).

When SOC is turned on, as shown in the right panel of Fig. 1(e), the valence band maximum is shifted away from the Γ point, inducing an open of indirect band gap. A parity exchange could be observed between the top of valence band and the bottom of conduct band at Γ, which is clearly induced by the SOC interaction. To understand the origin of the parity inversion, we analyzed the orbital character of the band structures near Fermi energy, shown in Fig. 1(e). It can be found that SOC interaction not only opens up a fundamental gap of 203 meV at Γ point near Fermi level, but also deforms the top valence band into two-humped shape with *s* dominated valley point between two 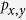 peaks. There is an exchange of *s*-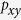 band component in the band structure, which is usually called band inversion and it is an important signal of topologically non-trivial. This could be further verified by using the Heyd-Scuseria-Ernzerhof (HSE) hybrid functional^21^, see Figure S1(a) in Supporting Materials (SM). As we can see, although the PBE calculations underestimate the band gap, it could still predict correctly the band inversion. As is pointed out by Zhang earlier^9^, for tin films in QSH states, HSE and PBE predict essentially the same nontrivial bulk gaps, as these bulk gaps are opened by SOC effect. Therefore, it is still feasible to predict qualitatively the topological properties in Sn-based films using PBE treatment. Due to the SOC, the bonding state of 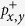 splits into two orbitals, with the higher orbital locating above Fermi level, while the lower one goes below 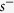 state, illustrated in stage III in Fig. 1(d). We noticed that Ma *et al.*^15^ has recently classified III-Bi bilayers into three types by their band structures, and our case belongs to their Type II according to the band inversion, which indicates that the value of the nontrivial band gap is smaller than the SOC strength (defined as the energy difference between two *p* orbitals after inducing of SOC).

In previous works by Zhang^9^, halogen decorated stanene is also reported as topological nontrivial. However, we notice their differences on the mechanism of band inversion, see Figure S2 of Supplementary Materials (SM). The orbital orders diverge in stage II, where SnCl has a lower 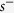 state than 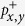. It belongs to type III in Ma’s classification^15^. The energetic differences between splitting 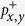 states in both cases are almost the same, indicating that the differences on functional groups have little effects on the SOC strength of tin atoms. Instead, the divergence on orbital orders is mainly caused by the energetic level of 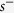 states, which has impacts mainly on the Sn-Sn σ bond. Compared to SnCl, the SnCN has a shorter Sn-Sn bond length, smaller lattice constant and lower buckled distance. The divergence occurs under Fermi level, therefore the final topological identifications come to the same.

### Topological properties and helical Edge states

The band gap induced by SOC, as well as the band inversion, both indicate the possibility of 2D TI phase. In order to identify 2D TI, we first calculate 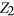 topological invariants^22^. When the structural inversion symmetry is present, 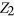 could be calculated simply following the approach proposed by Fu and Kane^23^, by checking the parities of Bloch wave function at four time-reversal-invariant momenta (TRIM) points 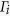
(one Γ point and three *M* points) in Brillouin zone (BZ). Accordingly, the 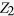 topological index *v* is established by


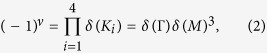


and


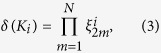


where *δ* is the product of parity eigenvalues at TRIM points, 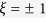 are the parity eigenvalues and *N* is the number of the occupied bands. *v* *=* 1 characterizes a QSH state, while *v* *=* 0 represents a topologically trivial band insulator. As all the bands are at least double degenerated under inversion symmetry, only occupied band with odd (or even) indices are taken into account in the productions above. By computing the product of half of the parity numbers for all occupied state at four time-reversal-invariant momentum (TRIM) points of BZ, we find the topological number *v* *=* 1, which suggests that it is topologically nontrivial QSH insulator.

**Figure 2:**
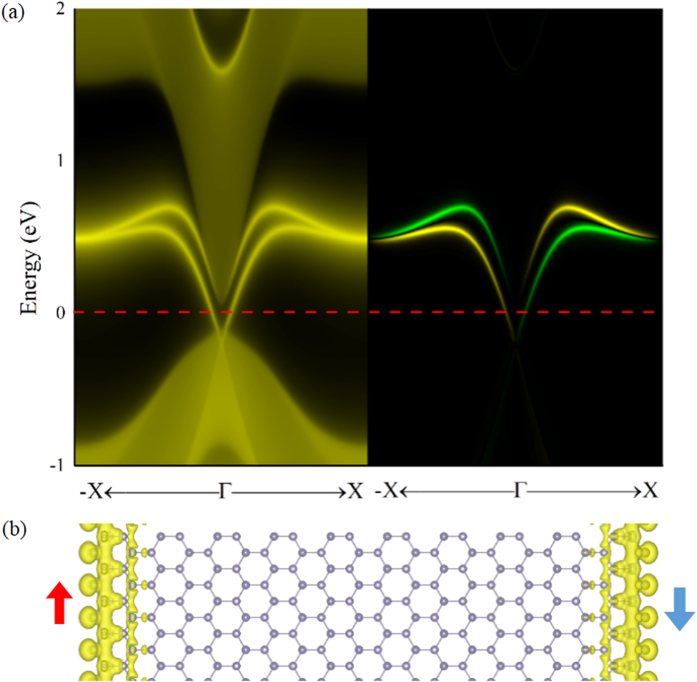
(**a**) Total (left panel) and spin (right panel) edge density of states for SnCN. In the spin edge plot, yellow/green lines denote the spin up/down polarization. The red dot line shows the Fermi level. (**b**) Real-space band decomposed charge density distribution from the edge states. The red/blue arrow denote the electrons with up/down spin.

In order to verify the existence of helical edge states with the spin-momentum locked by TRS, we try to illustrate the protected Dirac states by introducing edge on SnCN at ground state, see Fig. 2(a). The plot in the left panel shows the local density of states (LDOS) of a side-cut surface. A gapless edge states (bright lines in the figure) appear inside the band gap of bulk, cross at *X* (and −*X*), and connect the top of valence band and the bottom of conduction band. Further check on the spin components (yellow and green lines represent spin up and down respectively) are given in the right panel, which shows clearly that the pair of edge states are counter-propagating which carry opposite spin polarizations. The asymmetric spin polarization verified that SnCN is indeed a TI with the edge states protected by TRS. The real space band decomposed charge density of zigzag nanoribbon is plotted in Fig. 2(b), to confirm that the edge states are mainly contributed by the edge atoms in the ribbon.

Liu *et al.*^24^ emphasized the importance of chemical and structural edge modification in the practical application of 2D materials, therefore we also performed calculations on the band structure of Hydrogen-terminated zigzag ribbon of SnCN, see Figure S3 of SM. Dramatically, the original gapless edge states shift from the boundary to the center of BZ in *k*-space, and the Fermi velocity of the edge state is enhanced to be 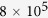 m/s, which is comparable with that of graphene 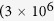 m/s). Meanwhile, the spin of the edge state shows a helical nature.

### Strain-induced topological transition and tunable gap under vertical electric fields

**Figure 3:**
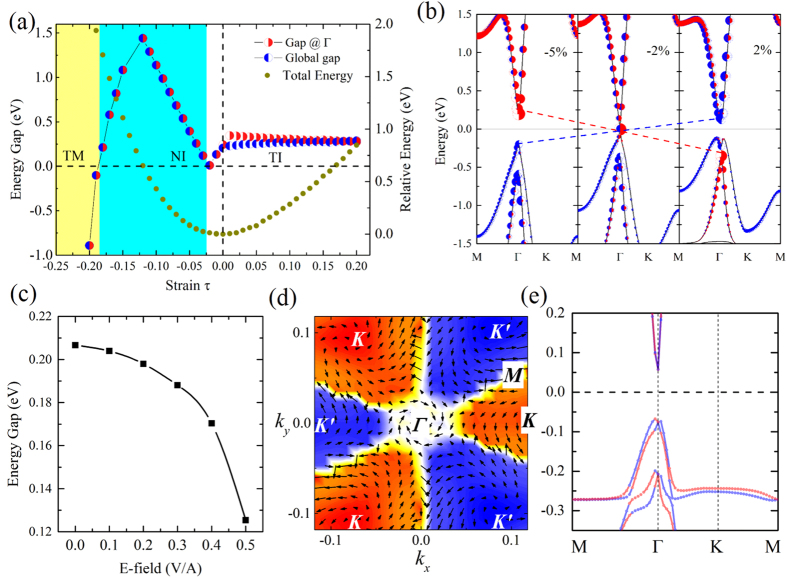
(**a**) Topological phase diagram of SnCN as a function of strains. The metal-NI (Normal insulator) and NI-TI (Topological insulater) transition occur at −18% and −2% of the strainless lattice constant, respectively. Red/blue half dots denote fundamental/inverted gap. Brown dots show the relative energy of structures under different strains. (**b**) Orbital-resolved band structure under different strains. Red/blue dots denote *s*/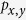 dominated orbitals. The size of dot denotes the weight of projection. (**c**) The energy gap as a function of extra vertical electric field. (**d**) Spin texture of the highest valence band of SnCN under electric field of 0.5 V/Å. Arrows refer to the in-plane orientation of spin, and the color background denotes *z* component of spin. (**e**) Spin-resolved band structure of SnCN under electric field of 0.5 V/Å. Red/blue lines denote bands with spin up/down polarization.

Hydrostatic tensile strain is applied then to modify the band structure of cyanogen decorated stanene. The tensile strain here is defined as 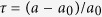, where 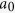 is the lattice constant of strainless state, compared to the compressed/stretched lattice *a*. As is shown in Fig. 3(a), with the variation of strains, two transitions occur in sequence, which are from metal to normal insulator (NI) and then TI. The system without strain is topologically nontrivial with an indirect bulk gap of 203 meV, as mentioned above. With the increase of strain, the difference between the global gap and that at Γ points decreases gradually, and system becomes direct band insulator under a strain of 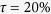.

The variation of band gap is more complicated when the system is compressed (*τ* < *0*). When *τ* decreases to −2%, the system becomes direct gap insulator with *v* = 1, and turns to be gapless eventually. With applications of further compression, the bulk gap grows instead, and of greater importance, a TI-NI transition arises under a strain of −3%. To illustrate the origin of this transition, the orbital-resolved band structures before and after the critical point are plotted in Fig. 3(b), which shows the variation of band components under the strains of –5%, −2% and 2%, respectively, with the red/blue dots denote the *s*/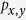 dominated bands. It is clear to see a band inversion at Γ point occurs during the transition. Accompanied by the change of component between the top of valence band and the bottom of conduction band, a parity swap leads the system to be topologically trivial. This is further verified by using HSE hybrid functional, see Figure S1(b).

With stronger compression applied on the system, the band gap of bulk has a linear increase and reaches the maximum under a strain of −13%. After that, the gap decreases rapidly and becomes negative when 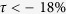, which indicates that an NI-metal transition occurs. Interestingly we find them to be topologically non-trivial again, labelled as topological metal (TM) in Fig. 3(a). These TM phases have bulk channels for the transports of electrons, along with edge spin channels.

Electric field is also a common and important way to tune band structures, which is considered to be convenient and clean. We try to apply electric fields vertical to the film (defined as *z* direction), and the variations of band gaps are illustrated in Fig. 3(c). We find that the band gap decreases with the increase of electric field strength, suggesting that it is an effective way to tune the band gaps.

To understand the effects of electric field on the band structures, we also plot the spin texture of the highest occupied band in BZ, as well as the spin resolved band structure, under a vertical electric field of 0.5 V/Å, see Fig. 3(d,e). The color map shows the *z* direction spin components along 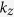, which illustrates a typical valley polarization, with clear boundaries between valleys, painted in white. We find that all the spin states near Γ point is perpendicular to momentums, together with the obvious spin splitting in Fig. 3(e), indicating a Rashba spin splitting. While at six *K* points, there are obvious spin vortex originating from *K* points and vanishing at the boundary lines along Γ−*M*.

### Topological Robustness against chemical impurities

**Figure 4:**
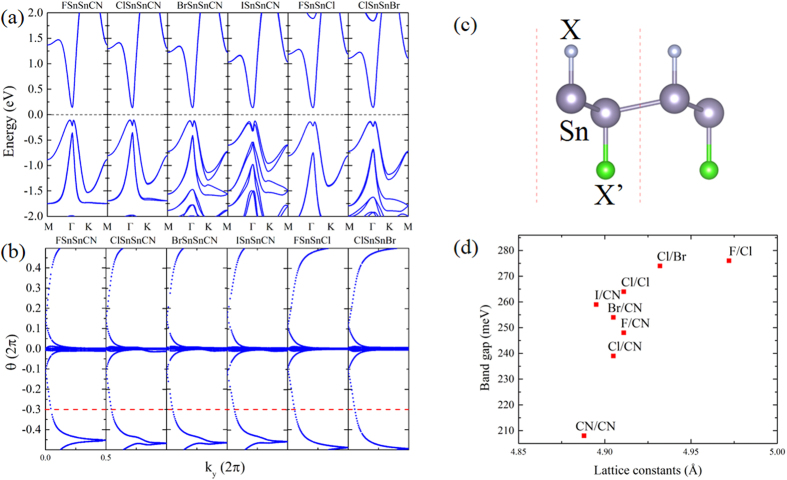
(**a**) Band structures of SnX_0.5_X′_0.5_. (**b**) Evolutions of Wannier centers for SnX_0.5_X′_0.5_ along 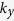. The evolution lines (blue dot lines) cross the arbitrary reference line (red dash line) parallel to 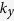 an odd number of times, yielding *v* = 1. (**c**) Crystal structure for SnX_0.5_X′_0.5_ unit cell from the side view. X and X′ represent different halogeno (F, Cl, Br and I) and cyano groups. (**d**) The calculated enegy gap for stanene decorated by different groups.

A big challenge to the synthesis of TI experimentally is the difficulty to obtain TI sample with high quality. Although not be reported experimentally yet, SnCN might be synthesized from halogen decorated stanene, through some cyanation reactions analogous to the case of aryl halides. Many cyanides are highly toxic, while recent researches^25^ have found some cheap and environment friendly cyanation reagent, such as K_4_[Fe(CN)_6_], which is considered as a secure source of CN for catalyzed cyanation. Topically, the yield of cyanation reaction of aryl halides is around 80–95%^25^, therefore, the final products would be mixtures of halogen and CN groups, which could be labeled as SnX_*x*_X′_1−*x*_ (X,X′ = cyanogen and halogen) family. Here we investigate and identify the topological properties of SnX_0.5_X′_0.5_ (X,X′ = cyanogen and halogen) as an example. Compare to SnCN, these mixed functionalized stanene have all honeycomb structure but a little larger lattice, see Fig. 4(d). The maximum expanding of lattice constant is less than 2%, indicating the good lattice matching.(Note that the topology of SnCN could preserve under expanding.) The band structures are given in Fig. 4(a). We can see significant band splitting at *k* points away from Γ in SnBr_0.5_(CN)_0.5_, SnI_0.5_(CN)_0.5_ and SnCl_0.5_Br_0.5_. It could be easily understood from the different abilities of CN and halogens to gain electrons. The bands near Fermi level are mainly contributed from Sn orbitals, therefore, the appearance of band splitting indicates the different effects of chemical groups on the Sn-Sn skeleton. The bonding energy of CN with Sn is between Sn-F and Sn-Cl, indicating that CN has a similar non-metallicity with F and Cl, while Br and I are metallically weak. So when presenting together with CN (e.g. in SnI_0.5_(CN)_0.5_), they would display different energetic behaviours, which leads to a more visible band splitting.

When the adsorbed atoms/groups on both sides of stanene are different, the inversion symmetry is absent, therefore the evaluation of topological index *v* by counting parities becomes impossible. Instead, we employ recently proposed Wilson loop methods^26^ based on the U (2N) non-Abelian Berry connection by Yu *et al.*^27^. In this method, each state of the *n*th occuped band is indexed by 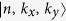, and a square matrix 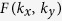 containing the overlap integrals


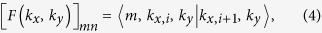


is defined. Then we calculate the complex unitary square matrix


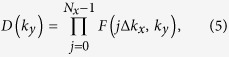


whose complex eigenvalues 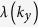 have phase angle *θ*. Here 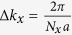 is the discrete spacing of 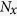 points along 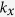 direction. Finally the 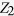 invariant *v* is calculated by counting the even or odd number of crossings of any arbitrary horizontal reference line with the evolution of *θ*, mod 2. The Wannier center evolutions of SnX_0.5_X′_0.5_ are shown in Fig. 2(b), and all of them have the arbitrary reference line crossing the evolution line an odd times, indicating clearly *v* = 1. This result could be generalized to SnX_*x*_X′_1−*x*_, which requires a detailed analyse on the orbital component. Similar to SnCN, the band gaps of SnX_*x*_X′_1−*x*_ are all induced by SOC effects from tin atoms. As is mentioned above, although SnCN and SnX (e.g. SnCl) have different orbital orders (*s* and 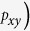, the divergence emerges underneath the Fermi level, which would not differ the final topological identifications. Therefore the mixture of decorated groups would not cancel the band inversion or change the topological order. It suggests the nontrivial topologies of SnX_*x*_X′_1−*x*_, and most important, their robustness against the chemical impurity of components.

It is meaningful in two aspects. First and the most important one is that the good topological properties could preserve under the impurity of chemical modification. It predicts that the topological properties of our system could preserve even with more than two kinds of chemically functional groups existing in the synthetic products. Therefore it is not requisite for the purification of functional groups and is economically efficient. And secondly, in practise, it is also feasible to tune the band gap by controlling the components of halogen/cyanogen in the synthesis of SnX_*x*_X′_*x*−1_. Meanwhile, the global band gaps in all cases are above 203 meV (5278K), suggesting the possible preservation of topological properties even at high temperature.

## Conclusions

In summary, we perform first-principles calculations to predict a novel QSH insulator of SnCN which has a gap up to 203 meV. The open of band gap is mainly contributed by SOC effect, which leads to inversion of band components and orbital parity. The stability of SnCN is confirmed by the phonon spectrum and large bonding energy, and its topological characteristic is identified by 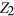 invariant and the helical edge states. The band gap can be engineered by the application of strains, vertical electric fields, and by the components of functional groups effectively. More interestingly, we notice that the topology of SnCN preserves when CN groups are partially replaced by halogen atoms, and it indicates that the topological properties of our system is robust against chemical impurities, which could make the synthesis economically efficient. Therefore, SnCN is an appropriate and flexible candidate material for spintronic devices.

## Methods

First-principle calculations are performed using plane wave basis Vienna ab initio simulation package (VASP)^28^,^29^,^30^,^31^. The projector-augmented wave (PAW)^32^,^33^ method is used to describe the electron-ion potential. The Perdew-Burke-Ernzerhof (PBE)^34^ form exchange-correlation potential approximate is employed with a 500 eV kinetic energy cutoff. As the bulk gaps in our system are opened by SOC effect, different functionals would give essentially the same predictions on topologies. Band structures are further verified using Heyd-Scuseria-Ernzerhof (HSE) hybrid functional^21^. Vacuum space of more than 20 Å is added to separate the structure studied with its periodic mirrors along *z* direction. We employ a *k*-point set generated by 11 × 11 × 1 Γ-centered Monkhorst-Pack mesh for both geometry optimization and self-consistent calculations. The atomic coordinates as well as the cell volume are fully relaxed, with forces on each atom converged underneath 0.01 eV/Å. The phonon spectra is calculated using a supercell approach within the phononpy code^35^.

In the whole process of the calculations, the advantages of Wannier functions are used with the Maximally localized Wannier functions (MLWFs)^36^ for the energy bands close to the Fermi level well constructed after the self-consistent electronic structure calculation. An iterative Green’s function method^37^,^38^ can be utilized to calculate the boundary states of a semi-infinite system based on MLWF, and is employed to observe the gapless edge state in our calculation.

## Additional Information

**How to cite this article**: Ji, W.- *et al.* Stanene cyanide: a novel candidate of Quantum Spin Hall insulator at high temperature. *Sci. Rep.* **5**, 18604; doi: 10.1038/srep18604 (2015).

## Supplementary Material

Supplementary Information
