# Supplementary material for: Stanene cyanide: a novel candidate of Quantum Spin Hall insulator at high temperature
Source: Sci Rep. 2015 Dec 21;5:18604. doi: 10.1038/srep18604 (PMC4685648; doi:10.1038/srep18604)
Supplement: Supplementary Information [file srep18604-s1.doc]

**Supplementary Materials**

**Stanene cyanide: a novel candidate of Quantum Spin Hall insulator at high temperature**

Wei-xiao Ji^1,*^ , Chang-wen Zhang^1,**^ Meng Ding^1^, Ping Li^1^ , Feng Li^1^ , Miao-juan Ren^1^ , Pei-ji Wang^1^ , Shu-jun Hu ^2^ and Shi-shen Yan ^2^

^1^ School of Physics and Technology, University of Jinan, Jinan, Shandong, 250022, P. R. China

^2^ School of Physics, State Key laboratory of Crystal Materials, Shandong University, Jinan, Shandong, 250100, P. R. China


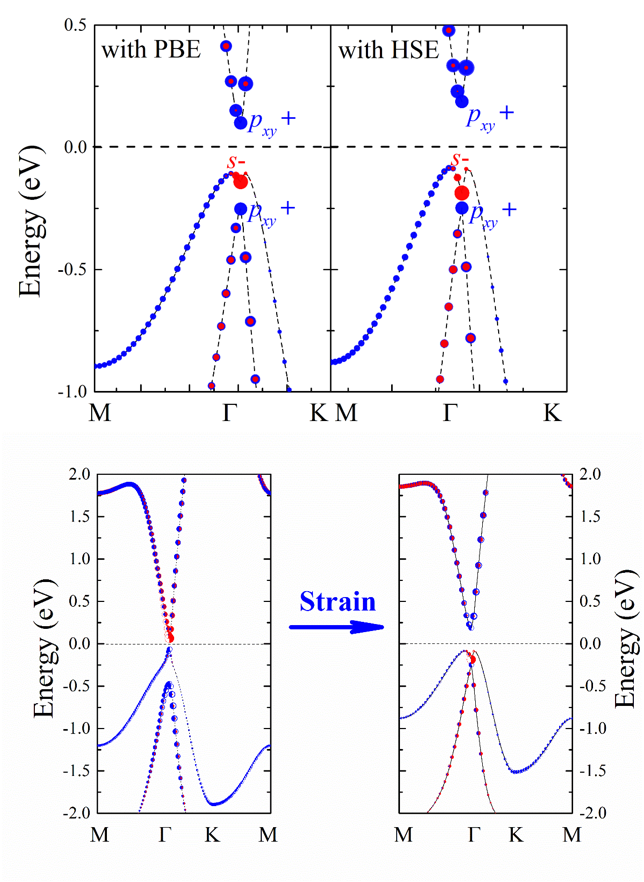


Figure S1: (a) The comparison between band structures of the strainless SnCN using PBE and HSE calculations. Red and blue dots denote orbital contributions from *s* and *p_xy_* of Sn atoms respectively, where +/- represent the parities of wavefunctions. (b) The band structures using HSE calculations before and after the NI and TI transition.


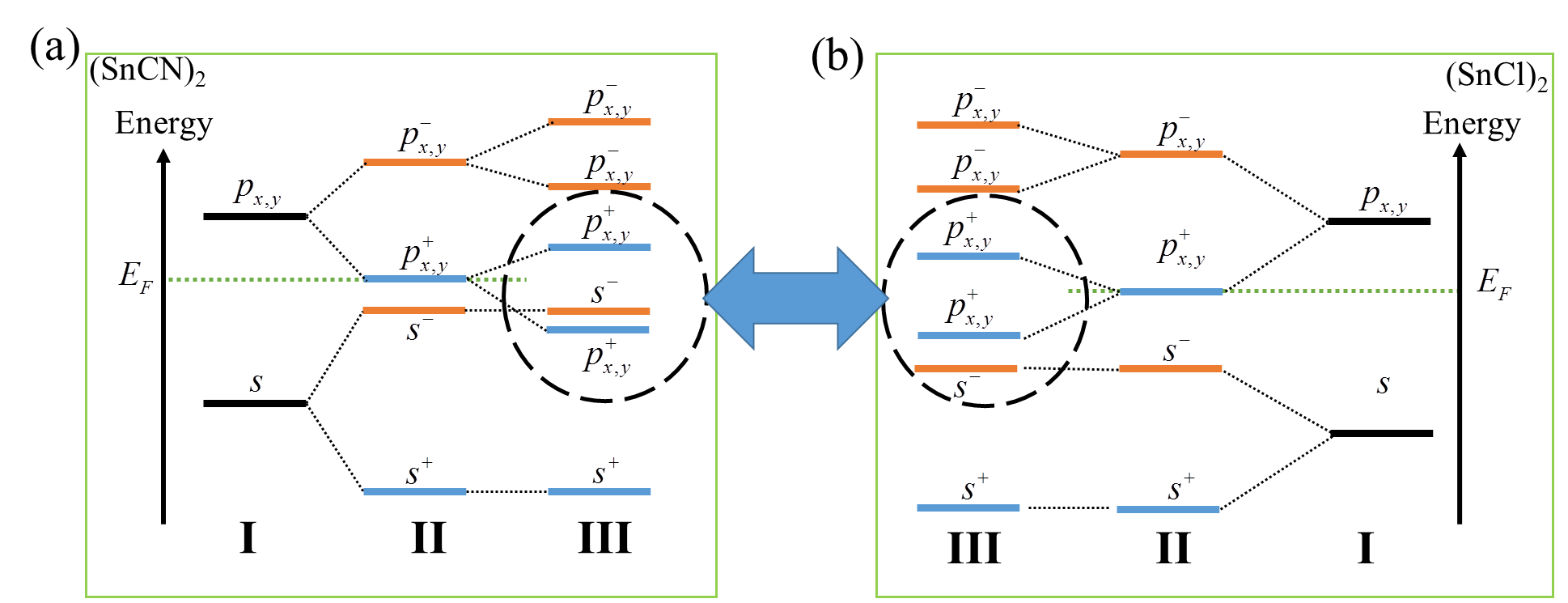


Figure S2: Schematic diagram of the evolution from atomic *s* and *p_xy_* orbitals of Sn in (a) SnCN and (b) SnCl_2_ into conduction and valence bands at Γ point. The three stages (I-III) represent the orbital orders with atomic potential, crystal field and SOC taken into account sequentially. The green dashed line denotes the Fermi energy EF.


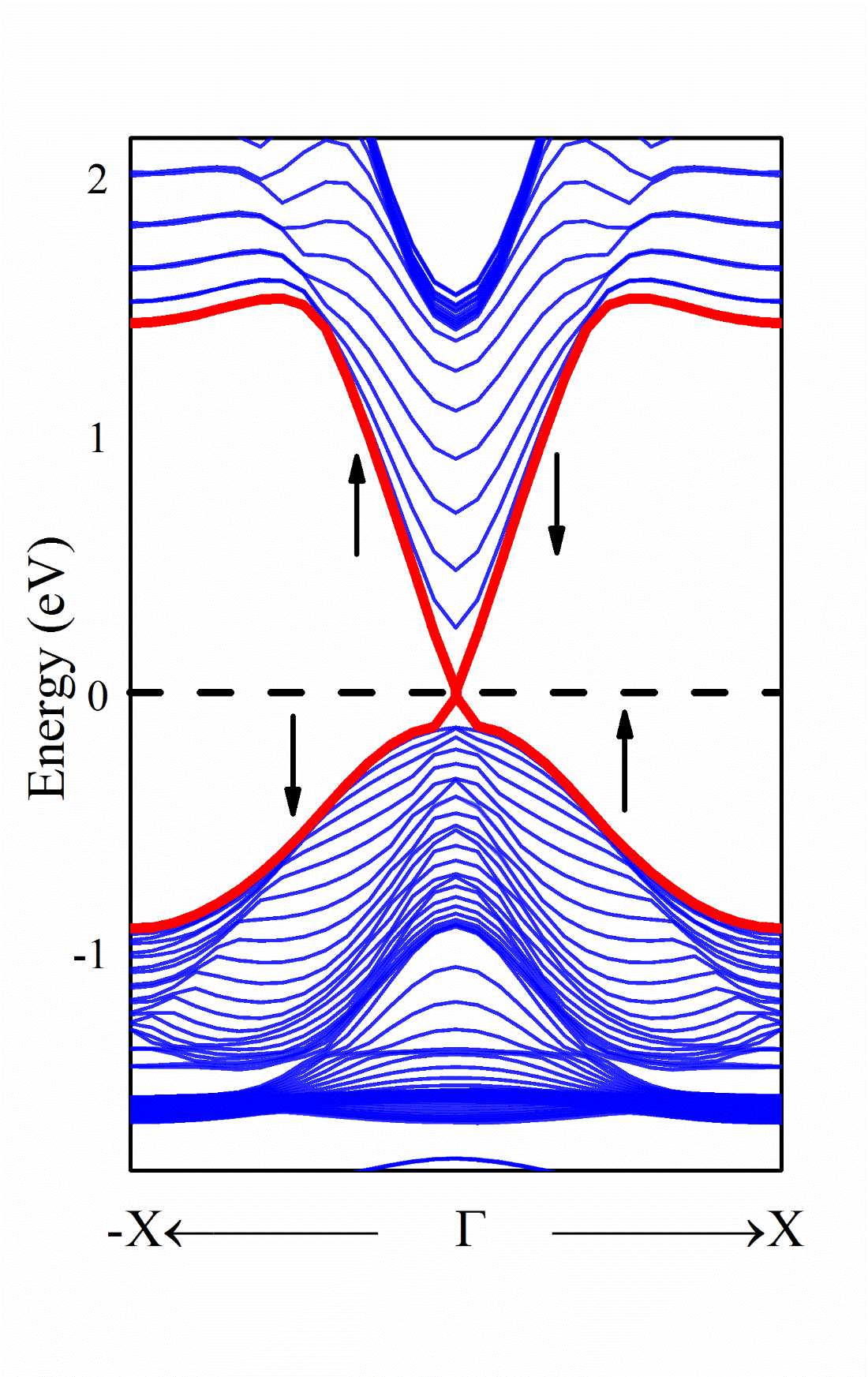


Figure S3: The band structure of H-terminated SnCN ribbon. The red lines denote the edge state, with the spin plotted with arrows. Only the spin components contributed from the left edge state are plotted, which are inverse to that of the right edge state.
